# Additions to the *Inocybe* sect. *Leptocybe* (Agaricales) in China: new species from tropical rainforests, new geographical distributions, and toxin detection

**DOI:** 10.3389/fmicb.2025.1540570

**Published:** 2025-05-20

**Authors:** Jia-Long Gao, Xiao-Peng Wu, Yu-Ling Zhou, Wen-Jie Yu, Yu-Guang Fan

**Affiliations:** ^1^Engineering Research Center of Tropical Medicine Innovation and Transformation of Ministry of Education, International Joint Research Center of Human-machine Intelligent Collaborative for Tumor Precision Diagnosis and Treatment of Hainan Province, Hainan Provincial Key Laboratory of Research and Development on Tropical Herbs, School of Pharmacy, Hainan Medical University, Haikou, China; ^2^Analysis and Test Center, Chinese Academy of Tropical Agricultural Sciences, Haikou, China; ^3^Hainan Academy of Inspection and Testing, Haikou, China

**Keywords:** Inocybaceae, new taxa, molecular phylogeny, taxonomy, toxin detection

## Abstract

*Inocybe* is a cosmopolitan genus of ectomycorrhizal fungi within the family Inocybaceae. Members of this genus are recognized as a group of toxic mushrooms linked to poisoning incidents worldwide. Clarifying this species diversity and toxin profiles within this genus is critical for both taxonomy and public health. In this study, we describe two newly identified species, *I. bicystidiata* sp. nov. and *I. microcarpa* sp. nov., based on phylogenetic analysis and morphological evidence. Phylogenetically, the two new species belong to *I.* sect. *Leptocybe*. *Inocybe bicystidiata* is characterized by nodulose basidiospores with saddle-shaped projections and the coexistence of thick-walled pleurocystidia and thin-walled paracystidia on the lamellar side. *Inocybe microcarpa* is characterized by very small basidiomata, spinose basidiospores with forked projections, absence of pleurocystidia, and thin-walled cheilocystidia. Ecologically, both of these new species occur in tropical rainforests dominated by *Parashorea chinensis*, which is considered as their presumed host. In addition, new geographic data are reported for three previously documented species of *I.* sect. *Leptocybe*, *I. acutata*, *I. juji*, and *I. peppa*, based on newly obtained specimens. Comprehensive ultra-performance liquid chromatography-mass spectrometry (UPLC–MS)/MS toxin screening revealed no detectable levels of muscarine, psilocybin, psilocin, bufotenine, or baeocystin in 10 examined species of sect. *Leptocybe*. This contrasts with the known toxin-producing *Inocybe* lineages, suggesting a divergent secondary metabolism in this clade.

## 1 Introduction

Inocybaceae Jülich (1982: 374) is a family of ectomycorrhizal fungi (Agaricales) commonly found in temperate and tropical forests, with over 1,050 described species across Africa, Asia, Europe, North America, Oceania, and South America (Matheny et al., 2020). The family has been revised based on the results of a recent phylogenetic study that divides the family into seven genera, with *Inocybe* (Fr.) Fr. (1863: 346) is the largest genus in the family (Matheny et al., 2020). Many species in the family contain fungal toxins such as muscarine, psilocybin, and phalloidin (Li et al., 2022; Zhang Y. Z. et al., 2022; Kosentka et al., 2013; Matheny et al., 2023), which usually lead to poisoning events (Li, H. J. et al., 2020, 2021, 2022, 2023, 2024; Xu et al., 2020; Deng et al., 2022; Chandrasekharan et al., 2020; Parnmen et al., 2021). Accordingly, the basic data on species diversity, geographical distribution, and their toxins are crucial for poison prevention and the utilization of this group of fungi (Deng et al., 2022). However, only a small proportion of Inocybaceae species have been assigned for toxin detection (Kosentka et al., 2013), and the toxin status of many taxa in the family remains under-recognized.

The genus *Inocybe* represents the most evolutionarily successful group, with over 1,000 documented species worldwide (Matheny and Kudzma, 2019). The members of *Inocybe* are characterized by small basidiomata, fibrillose to rimose pileus, smooth, nodulose to spinose basidiospores with a distinct apiculus, and usually thick-walled hymenial cystidia. However, *Inocybe* species collected from tropical forests have some peculiar features: thin-walled cheilocystidia or thin-walled pleurocystidia (Horak, 1979; Pradeep et al., 2016; Latha and Manimohan, 2017; Gao et al., 2024). The *I. alienospora* group was initially recovered in a LSU phylogeny with three species (*I. hydrocybiformis*, *I. lasseri*, and *I. stellata*), but the topography was not well supported (Horak et al., 2015). Subsequent studies expanded this clade to encompass Australian taxa (*I. alienospora*, *I. lasseroides*) and Indian taxa (*I. barbruka*, *I. kuruvensis*, *I. papiliformis*), formally designated as the *I. alienospora* clade (Pradeep et al., 2016; Latha and Manimohan, 2017; Matheny and Bougher, 2017). Our recent work has refined the phylogenetic framework of this group through the inclusion of specimens from China, leading to its formal classification as *I.* sect. *Leptocybe* (Gao et al., 2024). Concurrently, eight new species from temperate to tropical China and new distributions for certain species were reported (He et al., 2022; Gao et al., 2024). At present, 17 species are recognized within the sect. *Leptocybe*; however, several phylogenetically distinct lineages remain unresolved and necessitate taxonomic revision.

During a 2024 mycological survey in Xishuangbanna Tropical Rainforest National Park (Yunnan Province, China), two previously undescribed species of *I.* sect. *Leptocybe* were discovered. Integrated morphological examination and molecular phylogenetic analyses confirmed their taxonomic novelty. We provide detailed descriptions of these new species, supplemented with diagnostic illustrations and comparative discussions with allied taxa. Additionally, new geographic distributions of the previously documented species *I. acutata*, *I. juji*, and *I. peppa* were reported based on the recently obtained specimens. To better understand the toxic profiles of *I.* sect. *Leptocybe* in China, a targeted screening of toxins and quantitative analysis were performed using a comprehensive method of ultra-performance liquid chromatography-mass spectrometry (UPLC–MS/MS).

## 2 Materials and methods

### 2.1 Chemicals and reagents

Standards: Muscarine (catalog No. 0000045268) was purchased from Merck. Psilocybin (catalog No. 017013022YD04020200507) and psilocin (catalog No. 002005025TR00920200417) were obtained from Shanghai Yuansi Standard Science and Technology Co., Ltd. Bofotenine (catalog No. CFN91165) was sourced from Shanghai Keshun Biotechnology Co., Ltd. (Shanghai, China), and baeocystin (catalog No. B115315) was purchased from Toronto Research Chemicals. All standard solutions were stored at −20°C and protected from light. HPLC-grade acetonitrile and methanol were obtained from HuBei FTSCI BioTech Co., Ltd. (Wuhan, China), Acetic Acid and Ammonium acetate were obtained from Xilong Scientific Co., Ltd. (Shantou, China), and Shanghai Aladdin Biochemical Technology Co., Ltd. (Shanghai, China) Ultrapure water with electrical resistivity of 18.2 MΩ/cm and total organic carbon (TOC) < 3 ppb used in all experiments was produced by a Milli-Q water purification system (Millipore, Billerica, MA, United States).

### 2.2 Field sampling and morphological studies

Specimens were collected from Wangtianshu Scenic Area, a national nature reserve in Mengla County, Xishuangbanna Prefecture, Yunnan Province, China, with a tropical climate. In the field, ecological images were taken using a digital camera. The Basidiomata were documented while fresh, with color assignments based on the criteria set by Kornerup and Wanscher (1978). Subsequently, the specimens were dried overnight in an electric oven at 45°C and then sealed in plastic bags (Yu et al., 2020; Deng et al., 2021a, 2021b, 2022; Zhao et al., 2022; Hu et al., 2023). Following the study, the specimens were deposited in the Herbarium of the Changbai Mountain National Natural Reserve (ANTU), along with their corresponding FCAS (Fungarium of Changbai Mountain Academy of Sciences, FCAS) numbers.

Macromorphological features were documented from field notes and color photographs. Microscopic examinations were carried out using a light microscope. Mushroom tissues from the pileus, lamellae, and stipes were cut into thin sections by hand with the aid of a stereoscope (AV100–240 V). Dried specimens were sliced and rehydrated in a 5% potassium hydroxide (KOH) solution, and a 1% Congo Red solution was used when necessary. Basidiospores, basidia, hymenophoral trama, pleurocystidia, paracystidia, cheilocystidia, pileipellis/pileal trama, stipitipellis/stipe trama, and oily hyphae were examined and measured. For each specimen, side views of at least 100 mature basidiospores were measured, excluding the apiculus, using the format [a/b/c] to denote the measurement of “a” basidiospores from “b” individuals across “c” collections. Measurement data were presented as (d) e−h−f (g), where “d” and “g” represent the minimum and maximum values, respectively; “e” and “f” correspond to the values at the 5th and 95th percentiles when the data are ordered from smallest to highest; and “h” signifies the average value (Ge et al., 2021; Liu et al., 2021; Na et al., 2022). Furthermore, the roundness of spores was quantified using the length-to-width ratio (*Q*), which effectively differentiated between species. *Q*_m_ denotes the average *Q*-value, and *Q*_m_ ± SD indicates the average plus or minus the sample standard deviation (Ge et al., 2021).

### 2.3 DNA extraction, polymerase chain reaction, and sequencing

Three loci were identified from the samples in this study, including the rDNA internal transcribed spacer (ITS) region, 28S (LSU, large subunit of ribosomal DNA), and the second largest subunit of DNA-directed RNA polymerase II (*rpb2*). The genomic DNA was extracted using the NuClean Plant Genomic DNA Kit (ComWin Biotech, Beijing, China) and stored at −20°C. Polymerase chain reaction (PCR) was performed using the primer pairs ITS1-F/ITS4 for the ITS region (Gardes and Bruns, 1993), LR0R/LR7 for the 28S region (Vilgalys and Hester, 1990), and *rpb2*-6F/*rpb2*-7.1R for the *rpb2* region (Matheny, 2005). The standard PCR reaction mixture consisted of 9.5 μL water, 12.5 μL 2 × Taq Plus Master Mix (Dye) (CW0690L, ComWin Biotech, Beijing, China), 1 μL of each primer, and 1 μL template DNA. The PCR program consisted of an initial heating step of 5 min at 95°C for 4 min; then 35 cycles of denaturation at 94°C for 1 min, annealing at 53°C for 1 min and extension at 72°C for 1 min, with a final extension at 72°C for 8 min (Zhang M. et al., 2022). After amplification, the PCR products were sent to Sangon Biotech (Guangdong and Hainan) Ltd. for purification and sequencing. The sequencing results were analyzed using BioEdit v7.0.9.0 software (Hall, 1999) and assembled using SeqMan v7.1.0 within DNASTAR v7.1.0 (44.1) software (Burland, 2000). The newly generated DNA sequences were submitted to GenBank sequence database.^1^

### 2.4 Sequence alignment and phylogenetic analyses

For the phylogenetic analysis, validated sequences of *I.* sect. *Leptocybe* were retrieved from GenBank (Matheny and Moreau, 2009; Osmundson et al., 2013; Kaewgrajang et al., 2014; Horak et al., 2015; Latha and Manimohan, 2016, 2017; Matheny and Bougher, 2017; Bandini et al., 2020; He et al., 2022; Gao et al., 2024). *Nothocybe distincta* (K.P.D. Latha & Manim.) Matheny & K.P.D. Latha was used to root the phylogenetic tree (Latha and Manimohan, 2016). The alignment of the three partitions was performed using the MAFFT online tool^2^ with the E-INS-i iterative refinement strategy (Katoh et al., 2019). Sequence alignments were manually refined using BioEdit v7.0.9.0 (Hall, 1999). The three individual partitions (ITS, 28S, and *rpb2*) were concatenated into a single multiple sequence alignment using MEGA v5.02 (Tamura et al., 2011). Maximum likelihood (ML) analyses were performed using the IQ-TREE web server with 1,000 bootstrap replicates of ultrafast bootstrap resampling (Trifinopoulos et al., 2016). The optimal models for each partition in Bayesian Inference (BI) analyses were determined using MrModeltest v2.3 (Nylander, 2004). Finally, BI analyses were performed using MrBayes v3.2.7a (Ronquist et al., 2012), with the selected models applied to each partition. Four Markov chains were run, sampling every 100 generations. The first 25% of the trees were discarded after confirming that the average standard deviation of partition frequencies was less than 0.009 (Ronquist et al., 2012). Results were processed using FigTree v1.4.3 software^3^, with support values (with ML bootstrap proportions ≥70% and BI posterior probabilities ≥95 or < 95%) displayed on each branch. Phylogenetic results are ultimately displayed and annotated on the tvBOT website^4^ (Xie et al., 2023).

### 2.5 Sample preparation for toxin detection

Dried mushroom samples (10 mg) were ground into powder and transferred to a 2 mL centrifuge tube. Then, 2 mL methanol/water (70:30, v/v) was added to the mixture. The tubes were then vortexed for 30 min, placed in an ultrasonic bath (33 Hz, 25°C) for 30 min, and centrifuged at 12,000 rpm for 5 min at 4°C. Extraction solution was filtered through a 0.22 μm microporous membrane. Targeted screening was performed under the optimized UPLC-MS/MS detection conditions (Table 1). Extracts were prepared in the same way as described above and subjected to UPLC-MS/MS analysis. Working standards were prepared by mixing the stock standards with an acetonitrile/water solution (5,95, v/v). Calibration curves were generated with 1.0, 2.0, 5.0, 10.0, 20.0, 50,0, 100.0, and 200.0 ng/mL of the working standards solution using UPLC-MS/MS. Limit of detection (LOD) and limit of quantitation (LOQ) were calculated as the concentrations corresponding to signals that are 3 and 10 times the baseline noise’s standard deviation, respectively (Table 2).

**Table 1:** Mass spectrometry (MS) data for five mushroom toxins.

Compounds	Q1 Mass (Da)	Q3 Mass (Da)	DP (V)	CE (V)	Chromatographic condition	RT (min)
Muscarine	174.20	57.00	40.00	25.70	Mobile phase solvent: 10 mmol/L ammonium acetate aqueous solution (A), acetonitrile (B) Gradient elution: 0.5 → 3.5 min, 5 → 90% B; 3.5 → 5.0 min, 90% B; 5.0 → 7.0 min, 90 → 5% B; 7.0 → 10.0 min, 5% B	1.50
		97.00	40.00	25.40		1.50
Psilocybin	285.20	205.30	62.00	26.00		1.19
		58.10	62.00	53.00		1.19
Psilocin	204.90	160.10	45.00	23.00		2.73
		58.10	40.00	32.00		2.73
Bufotenine	205.00	160.10	69.00	19.00		2.74
		58.10	60.00	33.00		2.74
Baeocystin	271.10	160.00	37.00	21.60		0.99
		44.00	37.00	34.50		0.99

**Table 2:** The LOD, LOQ, recoveries, relative standard deviations (RSD), and precision of five mushroom toxins.

Toxins	LOD	LOQ	Recoveries	Precision
			(*n* = 6)	(*n* = 6), RSD
Muscarine	0.01	0.02	99.73%	1.77%
Psilocybin	0.01	0.02	95.45%	3.84%
Psilocin	0.02	0.04	92.95%	1.37%
Bufotenine	0.01	0.02	93.98%	2.94%
Baeocysitin	0.02	0.04	95.39%	2.00%

### 2.6 UPLC-MS/MS conditions

The liquid chromatography system consisted of two LC-30 AD pumps and a Shimadzu SIL-30 AC autosampler. An AB Sciex Triple Quad 6,500+ system (Applied Biosystems/MDS Analytical Technologies, Foster City, CA, United States) equipped with an electrospray ionization interface was used for mass spectrometric detection. An HSS T3 column (2.1 mm × 100 mm, 1.8 μm) (Waters, United States) was used as the separation column. The flow rate was 0.3 mL/min. The temperature of the analytical column was set at 40°C. The injection volume was 2 μL. Analyst software (v1.6) was used for detection, data acquisition, and processing. The specific UPLC parameters and mass spectral parameters of the fungal toxins are presented in Table 1.

## 3 Results

### 3.1 Molecular phylogeny

This study generated 22 new sequences (9 from ITS, 7 from 28S, and 6 from *rpb2*) and submitted them to GenBank (Table 3). The dataset included 53 taxa and 682 sites for the ITS partition, 52 taxa and 1,265 sites for the 28S partition, and 36 taxa and 613 sites for the *rpb2* partition. For the ML analyses, IQTREE automatically selected the following DNA substitution models: ITS (TVM + F + G4), 28S (HKY + F + I + G4), and *rpb2* (TNe + G4). ML phylogenetic analysis yielded a final log-likelihood value of −11828.001. MrModeltest identified the best-fitting models as GTR + G for the ITS and GTR + I + G for both the 28S and *rpb2* partitions. The BI phylogenetic analysis was completed after 120,000 generations, at which point the mean standard deviation of the split frequencies converged to 0.008840, and the adequate sample size (ESS) reached 365.42. The average potential scale reduction factor (PSRF) was 1.000.

**Table 3:** A list of taxa and specimen details used in molecular analyses.

Taxa	Voucher	Locality	GenBank accession number			References
			ITS	28S	*rpb2*	
*Inocybe acutata*	NJ4747	China	OR759137	OR760305	OR775214	Gao et al. (2024)
*I. acutata*	FYG4322	China	OR755906	n/a	n/a	Gao et al. (2024)
***I. acutata***	**FYG10441**	**China**	**PQ495596**	**n/a**	**n/a**	**This study**
***I. acutata***	**Y2482427**	**China**	**PQ495600**	**n/a**	**n/a**	**This study**
*I.* aff. *alienospora*	PBM3758	Australia	KP171107	KM197212	KM245973	Matheny and Bougher (2017)
*I.* aff. *hydrocybiformis*	DED8165	Thailand	GQ893018	GQ892973	n/a	Horak et al. (2015)
*I.* aff. *lasseroides*	PBM3786	Australia	KP171147	KP170926	KM245994	Matheny and Bougher (2017)
*I.* aff. *lasseroides*	TJB10466	Australia	KP171149	KP170928	KM245996	Matheny and Bougher (2017)
*I. alienospora*	PBM3743	Australia	KP171104	KM197209	KM245970	Matheny and Bougher (2017)
*I. alienospora*	REH9667	Australia	KP171105	KM197210	KM245971	Matheny and Bougher (2017)
*I. aprica*	FYG7640	China	OR755901	OR760197	OR775210	Gao et al. (2024)
*I. aurescens*	FYG2015387	China	OR755913	OR760276	OR775213	Gao et al. (2024)
*I. aurescens*	FYG2871	China	OR755902	n/a	OR775212	Gao et al. (2024)
*I. babruka*	CAL: 1344	India	KY440086	KY549116	KY553237	Latha and Manimohan (2017)
***I. bicystidiata***	**FYG10585**	**China**	**PQ422907**	**PQ422909**	**PQ429107**	**This study**
***I. bicystidiata***	**FYG10586**	**China**	**PQ422908**	**PQ422910**	**PQ429108**	**This study**
*I. carpinicola*	FYG6307	China	OP207874	OP207868	OP227086	He et al. (2022)
*I. carpinicola*	HK 0986	China	n/a	PP346378	PP356979	Gao et al. (2024)
*I. casuarinoides*	FYG8123	China	OR755909	OR759978	OR775206	Gao et al. (2024)
*I. casuarinoides*	FYG9895	China	OR975606	OR975624	PP356974	Gao et al. (2024)
*I. haikouensis*	FYG9868	China	OR975602	OR975620	PP356983	Gao et al. (2024)
*I. haikouensis*	FYG9893	China	OR975604	OR975622	PP356972	Gao et al. (2024)
*I. heteromorpha*	FYG5769	China	OR755900	OR759987	OR775207	Gao et al. (2024)
*I. heteromorpha*	FYG5769a	China	OR755910	OR760195	OR775208	Gao et al. (2024)
*I. hydrocybiformis*	ZT10077	Thailand	GQ893016	GQ892971	n/a	Matheny and Bougher (2017)
*I. hydrocybiformis*	ZT9879	Thailand	GQ893017	GQ892972	n/a	Horak et al. (2015)
*I. juji*	123	China	OR975596	OR975614	PP356982	Gao et al. (2024)
*I. juji*	653	China	OR975597	OR975615	PP356967	Gao et al. (2024)
***I. juji***	**FYG10450**	**China**	**PQ495595**	**PQ495602**	**n/a**	**This study**
*I. kuruvensis*	K(M) 191,734	India	KM924522	KM924517	KY553246	Latha and Manimohan (2016)
*I. lasseri*	MCA 1971	Guyana	n/a	EU569857	EU569856	Matheny and Moreau (2009)
*I. lasseroides*	PBM3749	Australia	KP171145	KP170924	KM245993	Matheny and Bougher (2017)
*I. lasseroides*	PBM3750	Australia	KP171146	KP170925	n/a	Matheny and Bougher (2017)
***I. microcarpa***	**FYG10587**	**China**	**PQ495594**	**PQ495601**	**PQ498472**	**This study**
*I. papilliformis*	CAL1372	India	KY440096	KY549126	n/a	Latha and Manimohan (2017)
*I. papilliformis*	CAL1374	India	KY440097	KY549127	n/a	Latha and Manimohan (2017)
*I. peppa*	NJ4118	China	OR975591	OR975610	PP356984	Gao et al. (2024)
*I. peppa*	NJ4117	China	OR975592	OR975611	PP356980	Gao et al. (2024)
***I. peppa***	**YZ2023101421**	**China**	**PQ495598**	**PQ495604**	**PQ498474**	**This study**
***I. peppa***	**YZ2023102842**	**China**	**PQ495597**	**PQ495603**	**PQ498473**	**This study**
***I. peppa***	**YZ2024042051**	**China**	**PQ495599**	**PQ495605**	**PQ498475**	**This study**
*I. perlucida*	DB20-8-16-33	Germany	MN803157	MN803157	n/a	Bandini et al. (2020)
*I. perlucida*	PBM4328	USA	MT228849	MT228849	n/a	Matheny and Lewis, unpublished
*I. pseudoasterospora*	STU: SMNS-STU-F-0901288	Italy	MN803152	MN803152	n/a	Bandini et al. (2020)
*Inocybe* sp.	TO-2011	Italy	JF908197	JF908197	n/a	Osmundson et al. (2013)
*Inocybe* sp.	FYG1146b	China	OR759138	OR760463	OR775215	Gao et al. (2024)
*Inocybe* sp.	MEL: 2382681	Australia	KP013044	KP013044	n/a	Bonito et al. Unpublished
*Inocybe* sp.	MEL: 2382696	Australia	KP012875	KP012875	n/a	Bonito et al. Unpublished
*Inocybe* sp.	130822MFBPL0312	China	MW554479	MW554479	n/a	Zhou, Unpublished
*I. stellata*	DED8060	Thailand	GQ893010	GQ892965	KM656107	Horak et al. (2015)
*I. stellata*	ECV3651	Thailand	GQ893007	GQ892962	KM656105	Horak et al. (2015)
*I. stellata*	ZT10097	Thailand	GQ893008	GQ892963	n/a	Horak et al. (2015)
*I. stellata*	ZT10123	Thailand	GQ893009	GQ892964	n/a	Horak et al. (2015)
*I. stellata*	CAL1369	India	KY440106	KY549136	KY553251	Latha and Manimohan (2017)
*Nothocybe distincta*	CAL 1310	India	KX171343	NG057278	KX171345	Matheny and Moreau (2009) and Latha and Manimohan (2016)
*N. distincta*	ZT 9250	India	n/a	EU604546	EU600904	Matheny and Moreau (2009) and Latha and Manimohan (2016)
Uncultured *Inocybe*	Ino6	Thailand	AB854674	AB854674	n/a	Kaewgrajang et al. (2014)

Specimens and their sequences obtained in the present study were in bold.

**Figure 1:**
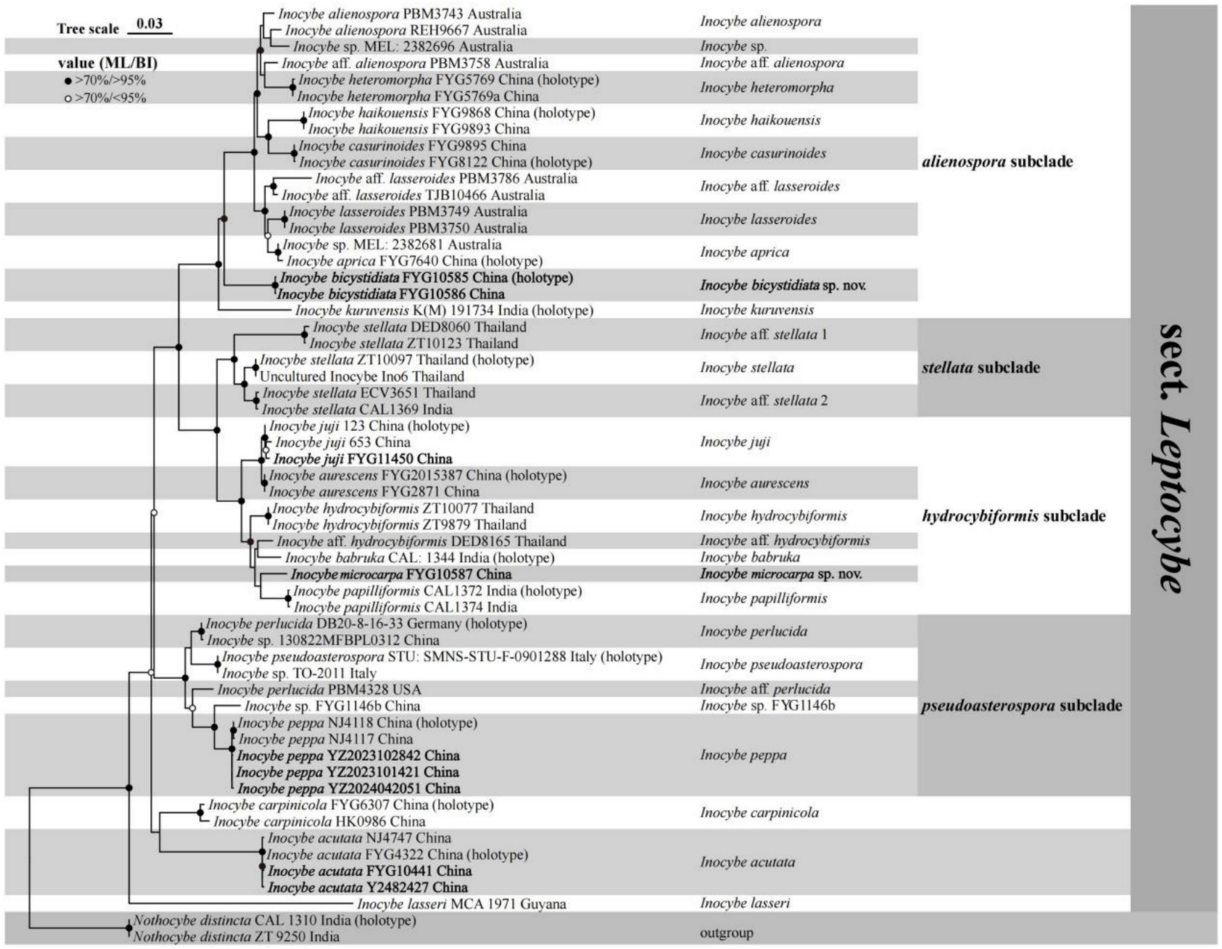
Phylogram generated by maximum likelihood (ML) and Bayesian inference (BI) analysis based on a combined dataset of nuclear genes (ITS, 28S, and *rpb2*). The tree is rooted with *Nothocybe distincta* (CAL1310 and ZT9250). Support values (ML-bp ≥ 70% and BI-pp ≥ 95% indicated by black circles; ML-bp ≥ 70% and BI-pp < 95% indicated by white circles centered in black) are shown at the nodes.

The phylogenetic results generated by ML and BI analyses have a similar topography, so only the ML tree is shown here. As shown in Figure 1, the two new species were placed in separate lineages in *I*. sect. *Leptocybe*. *Inocybe bicystidiata*, nested within the *alienospora* subclade, and was sister to most species except the basal taxon *I. kuruvensis* in this subclade. *Inocybe microcarpa* clustered with several tropical Asian taxa, *I. hydrocybiformis*, *I*. aff. *hydrocybiformis*, *I. barbruka*, and *I. papilliformis* in the *hydrocybiformis* subclade. Newly obtained specimens of *I. acutata*, *I. juji*, and *I. peppa* have been placed with their holotypes or authentic material in their respective lineages.

### 3.2 Taxonomy

***Inocybe acutata*** Takah. Kobay. & Nagas., Mycotaxon 48: 461 (1993)

**Remarks:** *Inocybe acutata* was originally described from Tottori, subtropical Japan, and subsequently found in China (Anhui, Zhejiang, and Jiangsu provinces). In 2024, we obtained three additional specimens from Jilin (temperate climate), Hubei (subtropical climate), and Guizhou (subtropical climate) provinces. *Inocybe acutata* is characterized by small and slender basidiomata, spinose basidiospores without saddle-shaped projections, absence of metuloid pleurocystidia, and thin-walled cheilocystidia. A detailed description and line drawings/color plates of *I. acutata* can be found in Kobayashi (1993) and Gao et al. (2024).

**Habitat and ecology:** Found in subtropical evergreen deciduous forests or temperate mixed deciduous and coniferous forests.

**Distribution:** China (Anhui, Zhejiang, Jiangsu, Jilin, Guizhou, and Hubei) and Japan (holotype).

**Newly collected specimens:** China. Jilin Province: Baishan City, Fusong County, Lushuihe Town, at 42°32′59″N, 128°00′39″E, alt. 649 m, 26 August 2024, leg. Tolgor Bau, Y2482427 (FCAS4073); Guizhou Province: Tongren City, Jiangkou County, Fanjingshan Nature Reserve, at 27°54′40″N, 108°28′32″E, alt. 1,600 m, 21 July 2024, leg. Y.-G. Fan and W.-J. Yu, FYG10441 (FCAS4074); and Hubei Province: Yichang City, Yiling District, in deciduous forest, alt. 1,086 m, 11 June 2024, Y.-P. Ge, L.-J. Wang, J.-W. Guo, G.-Y. Qiu, NJ 5065 (FCAS4078).

**Figure 2:**
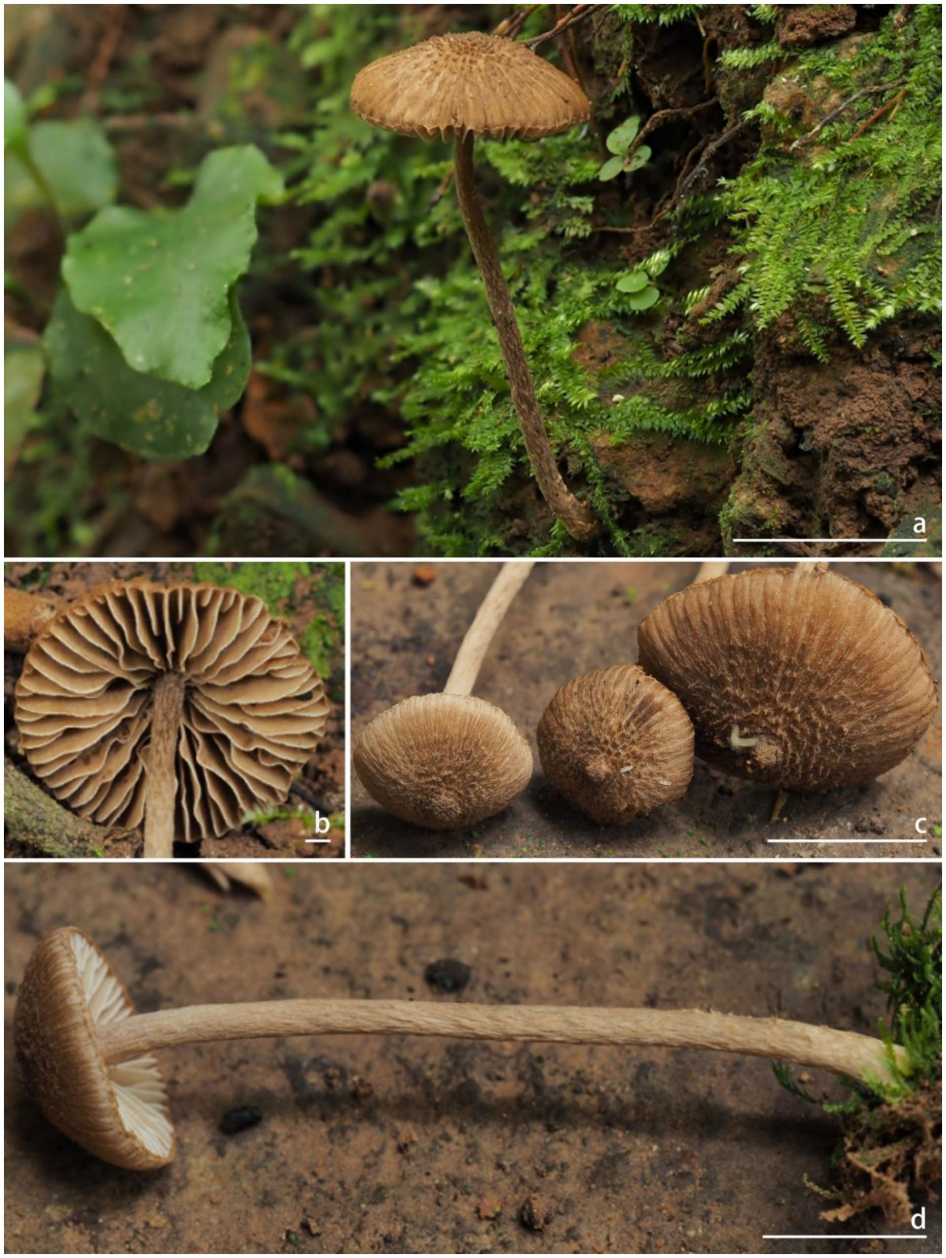
Basidiomata of *Inocybe bicystidiata*. **(a–c)** FYG10585 (FCAS4069, holotype); **(d)** FYG10586 (FCAS4070). Scale bars: **(a)** and **(c–d)** = 10 mm; **(b)** = 1 mm. Photos by Y.-G. Fan.

**Figure 3:**
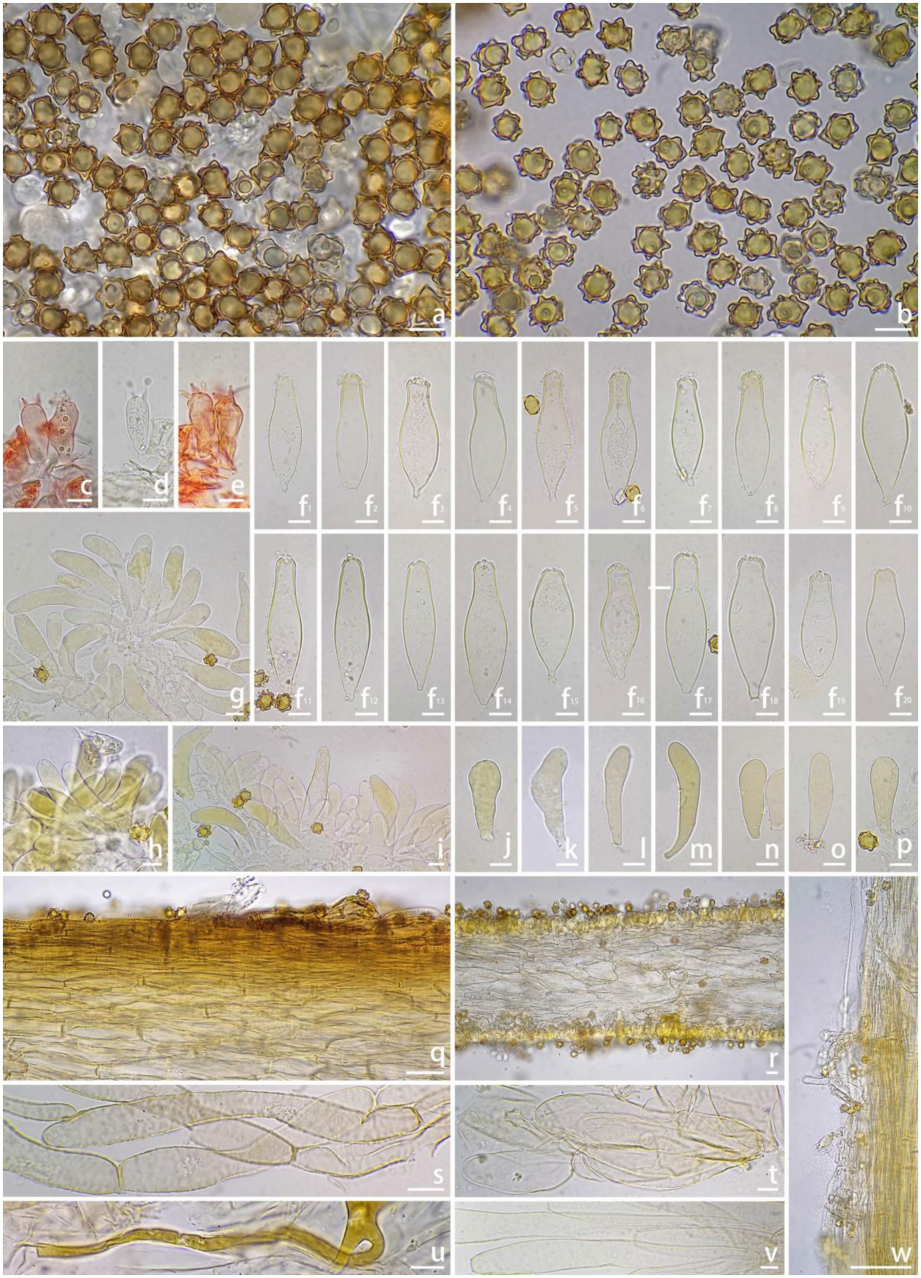
Microscopic features of *Inocybe bicystidiata* (FCAS4069, holotype). **(a,b)** Basidiospores; **(c–e)** Basidia; **(f**^**1**^**–f**^**20**^**)** Pleurocystidia; **(g–i)** Cheilocystidia; **(j–p)** Paracystidia in side of lamellae; **(q)** Pileipellis; **(r)** Hymenophoral trama; **(s)** Pileipellis hyphae; **(t)** Hymenophoral trama hyphae; **(u)** Oily hyphae; **(v)** Stipe trama hyphae; **(w)** Stipitipellis in the apex of stipe. Scale bars: **(a–v)** = 10 μm; **(w)** = 100 μm. Images by J.-L. Gao.

***Inocybe bicystidiata*** W.J. Yu, Y.G. Fan & J.L. Gao, sp. nov. Figures 2, 3 **Chinese name:** 双囊丝盖伞 (Double-cystidium Fiber Cap)

**Mycobank:** MB856828

**Etymology:** *bicystidiata* (L.), referring to the coexistence of thick-walled pleurocystidia and scattered thin-walled paracystidia on the lamellar side.

**Diagnosis:** *Inocybe bicystidiata* has slender basidiomata, thin- to thick-walled pleurocystidia, thin-walled yellowish-pigmented cheilocystidia, and a fungoid odor. Most similar to *I. kuruvensis*, but differs in the presence of thick-walled pleurocystidia and thin-walled paracystidia on the lamellar side.

**Holotype:** China. Yunnan Province: Xishuangbanna Dai Autonomous Prefecture, Mengla County, Xishuangbanna Tropical Rainforest National Park, Wangtianshu Scenic Spot, 21°37′21″N, 101°35′15″E, alt. 711 m, 28 July 2024, in tropical rainforest dominated by *Parashorea chinensis* Wang Hsie (Dipterocarpaceae), leg. Y.-G. Fan & W.-J. Yu, FYG10585 (FCAS4069), GenBank accession no: ITS (PQ422907); LSU (PQ422909) and *rpb2* (PQ429107).

**Description:** *Basidiomata* small and slender. *Pileus* 9–19 mm wide, hemispherical when young, then convex to planoconvex with a small pointed umbo or at times non-umbonate when matured; margin at first inrolled, then depressed to straight; cortina present in young specimens, often with brown analoid remnants from spore deposition; surface dry, dotted scaly around the disc, squamulose with finely recurved fibrils to mid-radius, radially fibrillose to rimulose with uneven streaks elsewhere; uniformly yellowish (4A2–4A4) when young, then yellowish (4B5–4C5) or yellowish-brown (5C4–5C5), brownish (5C6–5D6) to dark brown (5D6–5E6) toward the center when mature; veil remnants yellow (4C6–4D6) to pale brown (5C4–5C5). *Lamellae* adnexed, subdistant, 2–3 mm wide, alternately distributed with 4–6 tiers of lamellulae; white (4A1) or pale grayish-white (4A4–4B5) at first, yellowish-brown (5C4–5D5) to brown (5D5–5D6), edge pallid, indistinctly fimbriate. *Stipe* 40–52 × 1.2–2 mm, terete, central, solid, equal with a slightly enlarged base; surface dry, covered with a layer of veil remnants, appressed-fibrillose to silky smooth; yellow (4C6–4D6) to brown (5C3–5D4) when young, brown (5E4–5E5) when mature. *Context* fleshy in pileus, slightly yellow (4A4–4B4) with a pale brownish (5D6) tinge near the cuticle, 0.3 mm thick at mid-radius, up to 1.5 mm thick under the umbo; fibrillose and striate in stipe, yellowish (4C6–4D6) or slightly brownish (5D6). *Odor* fungoid.

*Basidiospores* [100/4/2], (7.0) 8.0–**9.44**–11.1 (12.2) × (5.9) 7.0–**8.31**–9.7 (11.2) μm, Q = (1.00) 1.00–**1.14**–1.29 (1.50), Q_m_ ± SD = 1.14 ± 0.099 with spines, (4.8) 5.0–**6.29**–7.2 (9.0) × (3.9) 4.2–**5.36**–6.7 (8.0) μm, Q = (1.00) 1.00–**1.18**–1.38 (1.55), Q_m_ ± SD = 1.18 ± 0.121 without spines; nodulose with spines that sometimes saddle-shaped projections or bifurcate, apiculus distinct, yellowish in 5% KOH, thick-walled, with yellow ovoid contents. *Basidia* 18–35 × 9–11 μm, subclavate to clavate, apex obtuse, bases usually tapered, with 4- or 2-sterigmata 2–9 μm length, colorless to slightly yellowish. *Pleurocystidia* 43*–*62 × 14–22 μm, abundant, mostly fusiform to utriform, apices rounded or obtuse, base usually tapering to a small pedicel, thick-walled, walls pale yellowish, up to 1.5 μm thick. *Paracystidia* on the lamellar side 32*–*61 × 9–15 μm, mostly clavate to broadly clavate, apices rounded or obtuse, base usually tapering to a small pedicel, colorless to slightly yellowish. *Cheilocystidia* 32–95 × 7–18 μm, abundant, subclavate to clavate, sometimes cylindrical or broadly clavate, apices rounded or obtuse, base tapered, thin-walled, colorless to pale yellowish. *Hymenophoral trama* 50–125 μm thick, subregular to regular, consisting of subinflated to inflated hyphae measuring 17–33 μm wide, colorless, smooth, thin-walled, wall slightly yellowish. *Pileipellis* a cutis, 35–48 μm wide, subregular to regular, yellowish-brown in mass, consisting of cylindrical hyphae measuring 12–15 μm wide, pale yellowish, walls slightly yellowish. *Pileal trama* 100–200 μm wide, regular, hyphae subinflated, colorless, 18–37 μm wide. *Stipitipellis* regular, hyphae cylindrical, 4–15 μm wide, encrusted, colorless. *Stipe trama* regularly arranged, composed of colorless, thin-walled, cylindrical hyphae 13–27 μm wide. *Caulocystida* not observed. *Oily hyphae* 3–6 μm wide, cylindrical, pale yellow to yellow, smooth, diverticulate, in hymenophoral trama. *Clamp connections* present in all tissues.

**Habitat and ecology:** Scattered on mosses in tropical rainforests dominated by *P. chinensis* (Dipterocarpaceae).

**Distribution:** Known from the type locality in Yunnan Province of China.

**Additional specimens examined:** CHINA. Yunnan Province: Xishuangbanna Dai Autonomous Prefecture, Xishuangbanna Tropical Rainforest National Park, Wangtianshu Scenic Spot, 21°37′21″N, 101°35′15″E, alt. 711 m, 28 July 2024, in tropical rainforest dominated by *P. chinensis* Wang Hsie (Dipterocarpaceae), leg. Y.-G. Fan & W.-J. Yu, FYG10586 (FCAS4070).

**Remarks:** *Inocybe bicystidiata* was recently found in Yunnan’s *P. chinensis*-dominated tropical rainforest. The non-umbonate pileus, featuring dotted, appressed to more or less raised squamulose, along with the slender continuate stipes, makes the new species impressive in the field. Microscopically, it has subglobose basidiospores with subconical or saddle-shaped nodules typically protruding about 2 μm long, thin-walled cheilocystidia, and thick-walled pleurocystidia together with scattered thin-walled paracystidia on the sides of the lamellae. The new species is phylogenetically placed in the *alienospora* subclade of *I.* sect. *Leptocybe* (Gao et al., 2024). This subclade includes *I. lasseroides*, *I. alienospora*, *I. kuruvensis*, and four recently described taxa from China, namely, *I. aprica*, *I. casuarinoides*, *I. haikouensis*, *I. heteromorpha.* Species in this subclade share thick-walled pleurocystidia and thin-walled cheilocystidia, usually with yellow pigments. In contrast to *I. bicystidiata*, the four recently described Chinese species exhibit brown to umber-brown and smaller basidiomata, appressed-fibrillose to appressed scaly pileus, apparently different outlines in the basidiospores, different associated plants (*Casuarina* or Fagaceae trees) and a geographical distribution in the Hainan province of China (Gao et al., 2024). The remaining three species are similar to the new species in having raised scales in the pileus, but *I. alienospora* has subumbonate pileus, more flanged or saddle-shaped nodules in the basidiospores and thicker walled pleurocystidia (Horak, 1979; Matheny and Bougher, 2017); *I. lasseroides* has umbonate pileus, ovoid–fusoid pleurocystidia with thicker walls (Matheny and Bougher, 2017); *I. kuruvensis* has dark brown pileus with erect scales, broadly fusiform pleurocystidia with thicker walls (Latha and Manimohan, 2017).

***Inocybe juji*** Y.G. Fan, Y.P. Ge & J.L. Gao, Mycology 15(4): 28 (2024)

**Remarks:** *Inocybe juji* was originally described from Anhui province, subtropical China. In 2024, we obtained an additional specimen from Hainan province (tropical climate). *Inocybe juji* is characterized by a dirty-yellow to brownish-yellow pileus, spinose basidiospores with saddle-shaped projections, and thin-walled and yellowish reflecting cheilo- and pleurocystidia. A detailed description and color plates of *I. juji* can be found in Gao et al. (2024).

**Habitat and ecology:** Scattered in subtropical evergreen broad-leaved forests dominated by fagaceous trees or in tropical cloud forests dominated by fagaceous trees.

**Distribution:** Anhui and Hainan Provinces in China.

**Newly collected specimens:** China. Hainan Province: Wuzhishan City, Nansheng Town, Wuzhishan station of Hainan Tropical Rain Forest National Park, at 109°40′43″E, 18°51′53″N, alt. 690 m, 2 August 2024, leg. J.-L. Gao, G.-H. Liu, and X. Chen, FYG10450 (FCAS4072).

**Figure 4:**
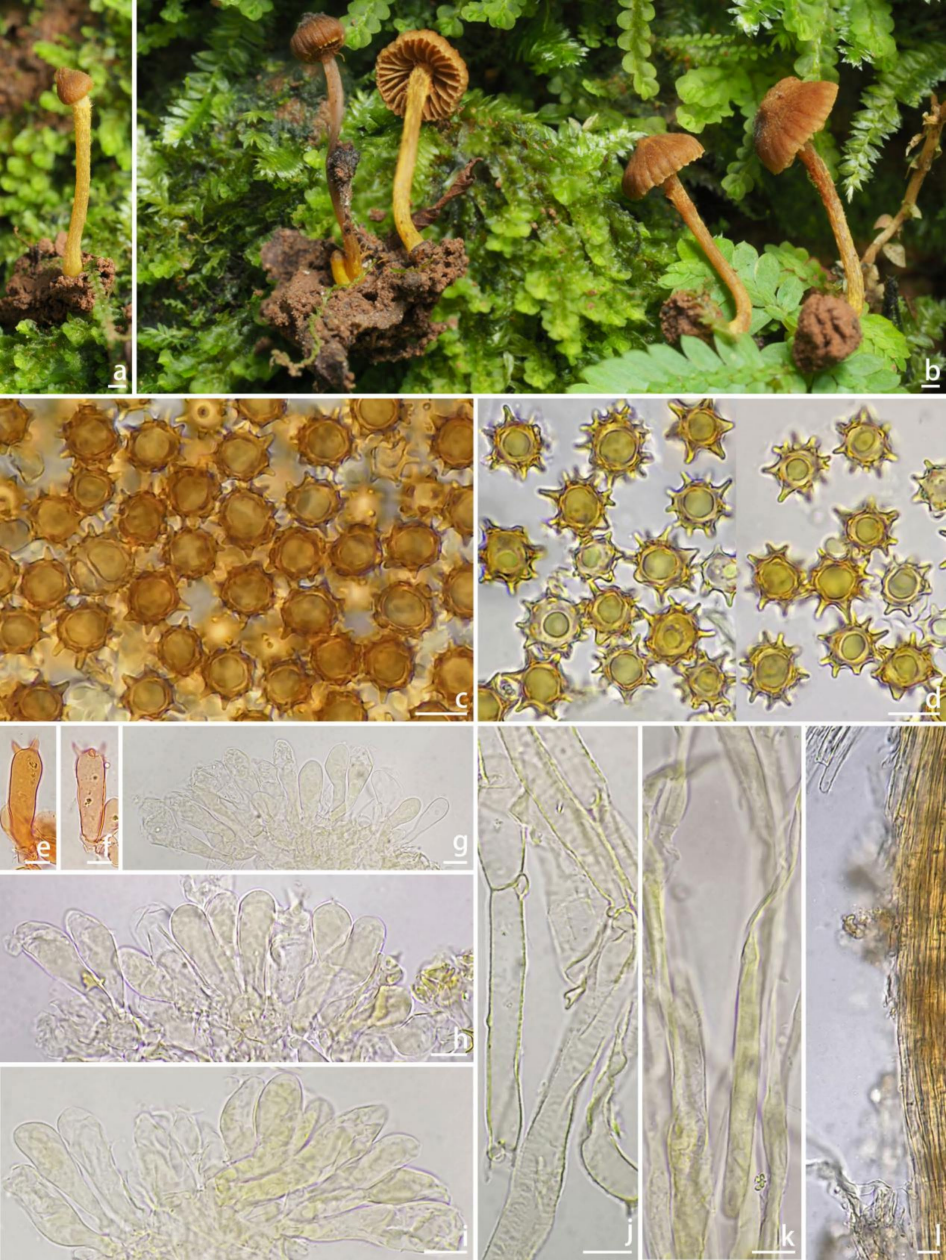
Basidiomata and microscopic features of *Inocybe microcarpa* (FCAS4071, holotype). **(a,b)** Basidiomata; **(c,d)** Basidiospores; **(e,f)** Basidia; **(g–i)** Cheilocystidia; **(j)** Pileipellis hyphae; **(k)** Stipitipellis hyphae; **(l)** Stipitipellis in the apex of stipe. Scale Bars: **(a,b)** = 10 mm; **(c–l)** = 10 μm. Images: **(a,b)** by Y.-G. Fan; **(c–l)** by J.-L. Gao.

***Inocybe microcarpa*** W.J. Yu, Y.G. Fan & L.J. Gao, sp. nov. Figure 4

**Chinese name:** 小果丝盖伞 (Tiny Fiber Cap)

**Mycobank: MB**856831

**Etymology:** *microcarpa* (L.), in reference to the conspicuously small basidiomata.

**Diagnosis:** *Inocybe microcarpa* has very small and slender basidiomata; grooved pileus, ginger-yellow veil remnants on stipe surface, subglobose to ovoid, spinose with simple or sometimes branched spines reaching up to 3.1 μm on basidiospores, and thin-walled, yellowish pigmented cheilocystidia. Most similar to *I. hydrocybiformis*, but differs in conspicuously smaller basidiomata, ginger-colored stipes, larger basidiospores, and shorter cheilocystidia.

**Holotype:** China. Yunnan Province: Xishuangbanna Dai Autonomous Prefecture, Xishuangbanna Tropical Rainforest National Park, Wangtianshu Scenic Spot, 21°37′21″N, 101°35′15″E, alt. 711 m, in tropical rainforest dominated by *P. chinensis* (Dipterocarpaceae), 28 July 2024, leg. Y.-G. Fan & W.-J. Yu, FYG10587 (FCAS4071), GenBank accession no: ITS (PQ495594); LSU (PQ495601); and *rpb2* (PQ498472).

**Description:** *Basidiomata* very small and slender. *Pileus* 4–5 mm wide, initially obtusely conical to campanulate, hemispherical to convex or broadly convex with a small subacute umbo, margin incurved when young, depressed to straight when mature, cortina present in young specimens; surface dry, glabrous or with finely fibrils toward the disc, radically fibrillose with grooves outward; uniformly brownish (5D4–5D5) to brown (6C4–6C5) when young, then yellowish brown (4C4–4D5) to brown (6C4–6C5), brownish (5C4–5C5) or yellowish brown (4C4–4D5) to yellowish (4B5–4B6) toward the center when mature. *Lamellae* adnexed, distant, 0.9–1 mm wide, alternately distributed with 2–3 tiers lamellulae; color initially yellowish brown (4C4–4D5) to yellow (4B4–4C5), then yellowish brown (4C4–4D5), yellowish (4A5–4B6) when mature, edge pale yellowish (4A3–4A4), not fimbriate. *Stipe* 10–12 × 0.8–1 mm, terete, central, solid, equal with a slightly enlarged base; surface dry, covered with a layer of yellowish (4B5–4C5) fibrils that from veil remnants at the apex; pale yellowish (4B3–4C4) to yellowish brown (4C4–4D5) when young, brownish (5C4–5C5) to darkly brownish (5E5–5E6) when mature. *Context* thin in pileus, translucent and pale yellowish in stipes. *Odor* not recorded.

*Basidiospores* [100/2/1], (9.9) 10.2–**11.55**–12.7 (13.1) × (8.7) 9.5–**10.60**–11.9 (12.2) μm, *Q* = (1.01) 1.02–**1.09**–1.20 (1.24), *Q*_m_ ± SD = 1.09 ± 0.052, subglobose to ovoid, spinose with simple or sometimes bifurcate spines up to 3.1 μm, pale yellowish in 5% KOH, thick-walled, sometimes with pale yellow ovoid contents. *Basidia* 27–38 × 10–15 μm, subclavate to clavate, apex obtuse, bases usually tapered, with 2- or 4-sterigmata 3–9 μm long, colorless to pale yellowish, sometimes golden yellowish. *Cheilocystidia* 28–47 × 8–12 μm, abundant, narrowly clavate to clavate, sometimes broadly clavate, apices rounded or obtuse, base tapered, thin-walled, colorless to pale yellowish, sometimes with golden yellowish pigments. *Hymenophoral trama* subregular to regular, consisting of cylindrical to subinflated hyphae 9–23 μm wide, colorless, smooth, thin-walled, walls slightly yellowish. *Pileipellis* a cutis, regular, yellowish to yellowish-brown in mass, consisting of cylindrical hyphae measured 5–10 μm wide, pale yellowish, smooth, thin-walled, walls pale yellowish. *Pileal trama* regular, hyphae cylindrical to subinflated, colorless, 10–15 μm wide. *Stipitipellis* regular, hyphae cylindrical, 4–9 μm wide, encrusted, smooth, colorless. *Caulocystida* not observed. *Oily hyphae* not observed. *Clamp connections* present in all tissues.

**Habitat and ecology:** Scattered on mosses in tropical rainforests dominated by *P. chinensis* (Dipterocarpaceae).

**Distribution:** Known from the type locality in Yunnan Province of China.

**Remarks:** *Inocybe microcarpa* is easily overlooked because of its very small basidiomata. It occurs on moss beds in tropical rainforests dominated by *P. chinensis*. The striped pileus and the ginger-colored fibrils on the stipes make this species conspicuous in the field. Microscopically, it has spinose basidiospores with bifurcate projections and thin-walled, yellow-pigmented cheilocystidia, and no pleurocystidia. Phylogenetically, *I. microcarpa* is placed in the subclade *hydrocybiformis* and tends to cluster with the lineage formed by *I. babruka*, *I. papilliformis*, and *I. hydrocybiformis*. These three species have similar spinose basidiospores with forked or saddle-shaped projections. However, *I. babruka*, described from Kerala (tropical India), has larger basidiomata, shorter projections in basidiospores, and longer cheilocystidia described as “gloeocystidia,” and a habitat near *Hopea ponga* trees (Dipterocarpaceae) (Latha and Manimohan, 2017); *I. hydrocybiformis*, described from Singapore and Malaysia and subsequently found in India, has larger basidiomata, shorter projections on average (up to 2.5 μm), longer cheilocystidia, and the presence of caulocystidia (Horak, 1979; Horak et al., 2015; Pradeep et al., 2016); *I. papilliformis* described from tropical India has larger basidiomata, an acute umbo in the pileus, significantly larger basidiospores measuring 15–19.5 × 14–18 μm, thick-walled pleurocystidia as metuloids, and a habitat on sandy soil under *H. parviflora* and *Vateria indica* (Dipterocarpaceae) (Pradeep et al., 2016; Latha and Manimohan, 2017).

***Inocybe peppa*** Y.G. Fan, Y.P. Ge, J.L. Gao & W.J. Yu, Mycology 15(4): 32 (2024)

**Remarks:** *Inocybe peppa* was originally described from Zhejiang, subtropical China. Here, we obtained four additional specimens from Sichuan (subtropical climate) and Shandong (warm temperate climate) provinces in 2023 and 2024. *Inocybe peppa* is characterized by small basidiomata, campanulate pileus, stellate basidiospores, and fusoid to broadly fusoid cheilo- and pleurocystidia. A detailed description and color plates of *Inocybe peppa* can be found in Gao et al. (2024).

**Habitat and ecology:** Scattered in subtropical evergreen broad-leaved forests.

**Distribution:** Zhejiang, Sichuan, and Shandong Provinces in China.

**Newly collected specimens:** China. Sichuan Province: Dazhou City, Xuanhan County, at 31°23′49″N, 107°34′42″E, alt. 500 m, 14 October 2023, leg. X.-M. Yang, YZ2023101421 (FCAS4076), 28 October 2023, leg. X.-M. Yang, YZ2023102842 (FCAS4075), 20 April 2024, leg. X.-M. Yang, YZ2024042051 (FCAS4077); Shandong Province, Tai’an, Dajinkou Town, Mount Tai, Yuquan Temple, at 36°18′15″N, 117°05′12″E, alt. 554 m, 20 July 2023, leg. Y.-P. Ge & Q. Na, HK1152 (FCAS4079), GenBank: ITS (PQ676244).

### 3.3 Method validation and toxin detection results

**Figure 5:**
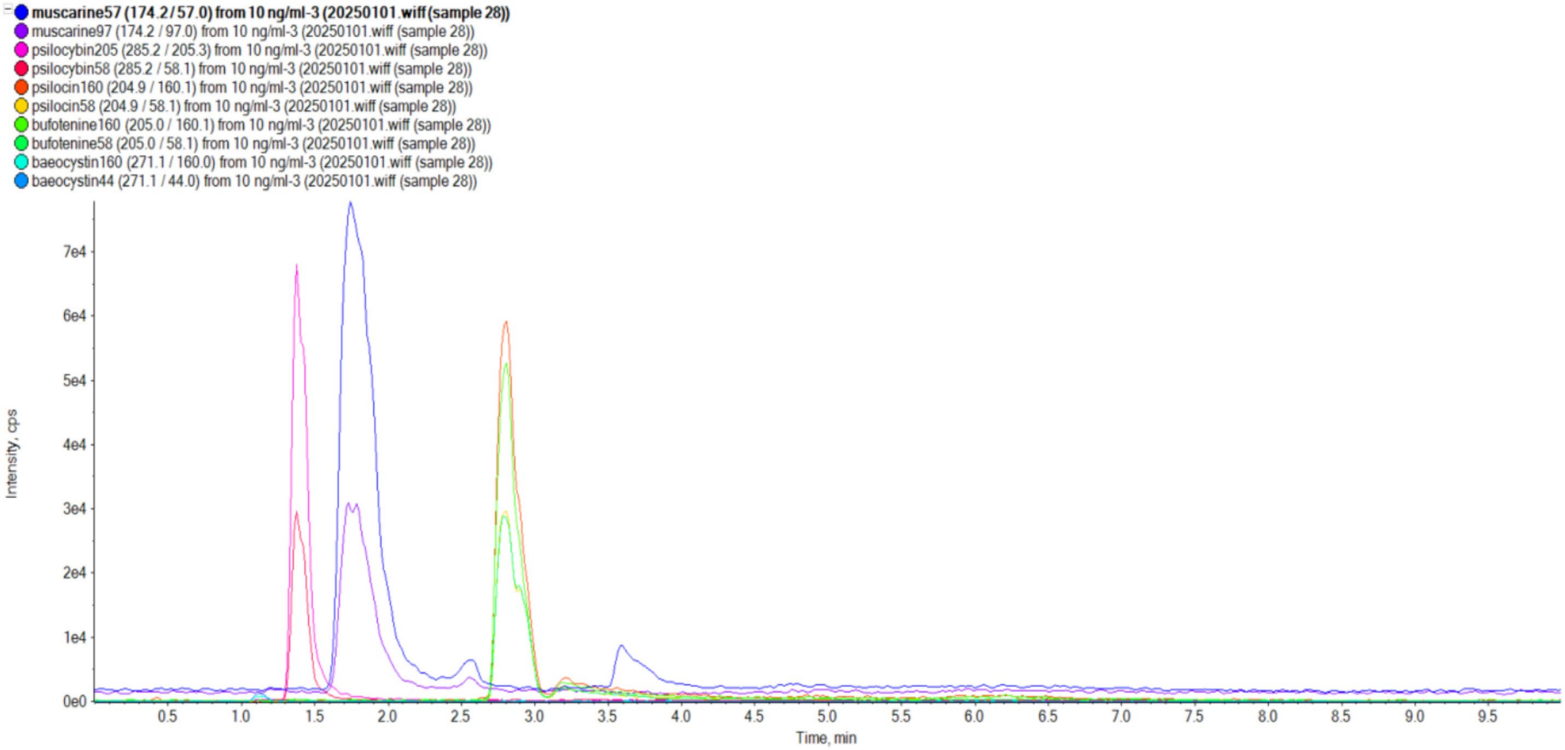
Standards of five mushroom toxins (muscarine, psilocybin, psilocin, bufotenine, and baeocystin).

UPLC-MS/MS analyses demonstrated robust method performance for toxin identification. Calibration curves of muscarine, psilocybin, baeocystin, psilocin, and bufotenine exhibited excellent linearity (*R*^2^ > 0.99; Figure 5). The precision of the analytical method was satisfactory, with a relative standard deviation of less than 5% for replicate measurements (Table 2). Method validation using *Lentinula edodes* spiked samples showed recoveries of 92.95%–99.73% and repeatability (RSD) within 2.60%–6.13% (Table 2), confirming the protocol’s suitability for quantifying these five toxins. All examined specimens of *I.* sect. *Leptocybe* (*n* = 32) underwent UPLC-MS/MS profiling. Targeted screening revealed no detectable levels of muscarine, psilocybin, psilocin, bufotenine, or baeocystin across 10 species within the section.

## 4 Discussion

The two new species, *I. bicystidiata* and *I. microcarpa*, were both discovered in tropical forests dominated by *P. chinensis*, a tree species of the Dipterocarpaceae family. *Parashorea chinensis* is also recognized as an iconic species in the tropical rainforests of China, but has a limited distribution in Yunnan and Guangxi provinces (Han et al., 2020). The tree species faces survival challenges due to changes in climate and native site conditions caused by human activities (Dai et al., 2017), and has been listed as a Class I endangered plant in China and by the International Union for Conservation of Nature (IUCN) (Dai et al., 2017; Li et al., 2004). The two new *Inocybe* species are hypothesized to be mycorrhizal partners of *P. chinensis*, but require further verification. In addition, new geographical distributions of *I. acutata*, *I. juji*, and *I. peppa* are reported based on recently collected specimens. Notably, *I. acutata* was discovered in Jilin Province, extending its range northward from subtropical southern China to temperate northeast China. Similarly, *I. peppa* was recorded in Shandong Province, a region characterized by a warm temperate monsoon climate. Additionally, *I. juji* was confirmed in the tropical montane cloud forests of Hainan.

Species of the *I.* sect. *Leptocybe* generally have small brown basidiomata, cortinatae and slender stipes, moderately crowded lamellae, thin-walled cheilocystidia and nodose basidiospores, usually with forked or saddle-shaped projections (Gao et al., 2024). However, notable exceptions occur within the section. For instance, the type species *I. acutata* exhibits simple spinose basidiospores and exclusively thin-walled pleurocystidia/cheilocystidia (Kobayashi, 1993); while *I. juji* and *I. aurescens* lack metuloids but retain thin-walled pleurocystidia and cheilocystidia. In this study, *I. bicystidiata* displays a unique combination: thick-walled pleurocystidia coexisting with thin-walled paracystidia on lamellar sides (Figure 3). While its paracystidia resemble those of *I. acutata*, the latter species completely lacks lamellar metuloids. Re-examination of Chinese *I. acutata* specimens revealed that its purported “paracystidia” structurally resemble basidioles, being nearly confluent with the hymenium layer. These elements can nevertheless be distinguished from true basidioles by their oily cytoplasmic inclusions and distinct subapical pigmentation in 1% Congo Red (Gao et al., 2024). Contrasting cystidial patterns are observed in the *hydrocybiformis* subclade: *I. hydrocybiformis* was originally described as lacking pleurocystidia (Horak, 1979), yet Thai specimens showed scattered thin-walled pleurocystidia resembling cheilocystidia (Horak et al., 2015); *I. papilliformi*s initially reported both metuloid cheilo- and pleurocystidia, but subsequent studies found no pleurocystidia (Latha and Manimohan, 2017). *Inocybe microcarpa* in our study showed no discernible thin-walled elements distinct from basidia/basidioles on lamellar sides.

Muscarine and psilocybin are the primary toxins in *Inocybe* fruiting bodies (Stijve et al., 1985; Stijve and Kuyper, 1985; Kosentka et al., 2013; Xu et al., 2020; Li, S. N. et al. 2021). Secondary psychotropic compounds (e.g., baeocystin and psilocin) and amatoxins have been sporadically reported in select species (Semerdzieva et al., 1986; Gartz, 1987; Kosentka et al., 2013; Ren et al., 2016). Despite the genus’ high species diversity, toxin profiles remain understudied, with only a limited subset of taxa analyzed to date. A literature review identified 13 *Inocybe* species testing negative for both muscarine and psilocybin: *I. appendiculata*, *I. fraudans*, *Inocybe* aff. *fraudans*, *I. godeyi*, *I. grammata* (= *I. albodisca*), *I. granulosipes*, *I. incarnata*, *I. luteifolia*, *I. nigrescens*, *I. subexilis*, *I. tahquamenonsis*, *I. viscata*, and *I. xanthomelas* (Kosentka et al., 2013).

Notably, our UPLC-MS/MS analyses detected none of the five targeted toxins (muscarine, psilocybin, psilocin, bufotenine, baeocystin) across the 10 examined species of *I.* sect. *Leptocybe* (Table 4). These species represent five of the seven major phylogenetic lineages within the section (Figure 1), though materials from the neotropical *I. lasseri* and the *I. stellata* subclade were unavailable for testing. The newly described *I. microcarpa* was excluded from toxin screening due to insufficient biomass.

**Table 4:** Information and weighing details of analyzed mushroom specimens.

Taxa	Specimen voucher	Weight (g)	Taxa	Specimen voucher	Weight (g)
*I. acutata*	FYG10441	0.0036	*I. juji*	119	0.0121
	NJ4119	0.0009		147	0.0101
	NJ4747	0.0030		123	0.0100
*I. aurescens*	FYG2015387	0.0104		653	0.0104
*I. carpinicola*	HK0985	0.0100		187	0.0100
	HK0987	0.0101		180	0.0113
	HK0986	0.0100		FYG11450	0.0102
*I. casuarinoides*	FYG8120	0.0107	*I. peppa*	NJ4118	0.0050
	FYG8122	0.0101		NJ4117	0.0113
	FYG8123	0.0128		YZ2024042051	0.0104
	FYG9871	0.0104	*I. aprica*	FYG9907	0.0107
*I. haikouensis*	FYG9866	0.0107		FYG9908	0.0108
	FYG9867	0.0112		FYG7640	0.0108
	FYG9868	0.0111		FYG7641	0.0053
	FYG9870	0.0102	*I. bicystidiata*	FYG10585	0.0108
*I. heteromorpha*	FYG5769	0.0108		FYG10586	0.0106

Muscarine production was considered an ancestral trait in the *Inocybe* s.s.-*Pseudosperma*-*Nothocybe* clade, but exhibits multiple evolutionary losses (Kosentka et al., 2013). This phylogenetic plasticity complicates definitive toxin status attribution at the sectional level. Consequently, the absence of detected toxins in *I.* sect. *Leptocybe* requires validation through expanded sampling, particularly for unscreened lineages (e.g., *stellata* subclade) and chemically uncharacterized species.

## Data Availability

The datasets presented in this study can be found in the online repository https://www.ncbi.nlm.nih.gov/genbank/ and the accession numbers are mentioned in Table 3.

## Footnotes

^1^ https://www.ncbi.nlm.nih.gov/genbank/

^2^ https://mafft.cbrc.jp/alignment/server/

^3^ http://tree.bio.ed.ac.uk/software/fgtree/

^4^ https://www.chiplot.online/tvbot.html
